# Diabetic Foot Ulcer Classification Models Using Artificial Intelligence and Machine Learning Techniques: Systematic Review

**DOI:** 10.2196/69408

**Published:** 2025-09-24

**Authors:** Manuel Alberto Silva, Emma J Hamilton, David A Russell, Fran Game, Sheila C Wang, Sofia Baptista, Matilde Monteiro-Soares

**Affiliations:** 1 USF Sanus Carandá, ULS Braga Braga Portugal; 2 Faculty of Medicine University of Porto Porto Portugal; 3 Department of Endocrinology and Diabetes Fiona Stanley Hospital Murdoch Australia; 4 University of Western Australia School of Medicine Fiona Stanley Hospital Murdoch Australia; 5 Leeds Institute of Clinical Trials Research University of Leeds Leeds United Kingdom; 6 Leeds Vascular Institute Leeds Teaching Hospitals NHS Trust Leeds United Kingdom; 7 University Hospitals of Derby and Burton NHS Foundation Trust Derby United Kingdom; 8 Division of Dermatology Department of Medicine University of Toronto Toronto, ON Canada; 9 Women’s College Hospital Toronto, ON Canada; 10 MEDCIDS—Departamento de Medicina da Comunidade Informação e Decisão em Saúde Faculty of Medicine University of Porto Porto Portugal; 11 CINTESIS@RISE—Center for Health Technology and Services Research Faculty of Medicine University of Porto Porto Portugal; 12 CUF Porto Hospital Porto Portugal; 13 Portuguese Red Cross Health School Lisbon Lisbon Portugal; 14 Cross I&D Lisbon Portugal

**Keywords:** artificial intelligence, diabetic foot, classification, machine learning, prognosis.

## Abstract

**Background:**

Diabetes-related foot ulceration (DFU) is a common complication of diabetes, with a significant impact on survival, health care costs, and health-related quality of life. The prognosis of DFU varies widely among individuals. The International Working Group on the Diabetic Foot recently updated their guidelines on how to classify ulcers using “classical” classification and scoring systems. No system was recommended for individual prognostication, and the group considered that more detail in ulcer characterization was needed and that machine learning (ML)–based models may be the solution. Despite advances in the field, no assessment of available evidence was done.

**Objective:**

This study aimed to identify and collect available evidence assessing the ability of ML-based models to predict clinical outcomes in people with DFU.

**Methods:**

We searched the MEDLINE database (PubMed), Scopus, Web of Science, and IEEE Xplore for papers published up to July 2023. Studies were eligible if they were anterograde analytical studies that examined the prognostic abilities of ML models in predicting clinical outcomes in a population that included at least 80% of adults with DFU. The literature was screened independently by 2 investigators (MMS and DAR or EH in the first phase, and MMS and MAS in the second phase) for eligibility criteria and data extracted. The risk of bias was evaluated using the Quality In Prognosis Studies tool and the Prediction model Risk Of Bias Assessment Tool by 2 investigators (MMS and MAS) independently. A narrative synthesis was conducted.

**Results:**

We retrieved a total of 2412 references after removing duplicates, of which 167 were subjected to full-text screening. Two references were added from searching relevant studies’ lists of references. A total of 11 studies, comprising 13 papers, were included focusing on 3 outcomes: wound healing, lower extremity amputation, and mortality. Overall, 55 predictive models were created using mostly clinical characteristics, random forest as the developing method, and area under the receiver operating characteristic curve (AUROC) as a discrimination accuracy measure. AUROC varied from 0.56 to 0.94, with the majority of the models reporting an AUROC equal or superior to 0.8 but lacking 95% CIs. All studies were found to have a high risk of bias, mainly due to a lack of uniform variable definitions, outcome definitions and follow-up periods, insufficient sample sizes, and inadequate handling of missing data.

**Conclusions:**

We identified several ML-based models predicting clinical outcomes with good discriminatory ability in people with DFU. Due to the focus on development and internal validation of the models, the proposal of several models in each study without selecting the “best one,” and the use of nonexplainable techniques, the use of this type of model is clearly impaired. Future studies externally validating explainable models are needed so that ML models can become a reality in DFU care.

**Trial Registration:**

PROSPERO CRD42022308248; https://www.crd.york.ac.uk/PROSPERO/view/CRD42022308248

## Introduction

Diabetes is a rapidly growing disease. Since 2000, the prevalence of diabetes has more than tripled, reaching, in 2021, 10.5% of the adult population in the world [1]. The increase in diabetes prevalence is associated with the rise of its related complications [2]. Diabetes-related foot ulceration (DFU), defined as a break in the skin of the foot that involves at least the epidermis and part of the dermis [3], is the most commonly recognized complication affecting the lower extremities.

The risk of a person with diabetes developing a DFU across their lifetime is around 19%-34% [4]. Approximately 20% of people who develop a DFU will require lower extremity amputation (LEA) [4], and 10% will die within 1 year of their first DFU diagnosis [5,6]. In the United States, foot complications contribute to US $273 billion in direct costs and $90 billion in indirect costs [7]. Apart from the impact of a DFU on mortality and health care costs, people with DFUs also have a significantly lower health-related quality of life [8].

The evaluation and prognosis of a DFU vary considerably according to person, limb, and ulcer-related characteristics. For that reason, classification and scoring systems were developed to create groups of patients with similar characteristics for whom similar levels of care would apply. Furthermore, they can be used to communicate wound and person-related characteristics between professionals, estimate an individual’s prognosis, help in clinical practice decision-making, and audit and comparison of populations.

A systematic review from the International Working Group on the Diabetic Foot (IWGDF) in 2023 found 28 different classification and scoring systems for DFUs [9]. As no gold standard exists, each system should be used according to the intended purpose, available resources, expertise, and clinical setting. In the IWGDF 2023 updated guidelines [10], no classification or scoring system was recommended for individual prognostication.

Expert opinion and conventional statistical methods, such as linear regression and other generalized linear models, have been used to develop classifications to help predict clinical outcomes in people with DFU [11,12]. However, these methods lack detail and do not capture the complex nonlinear relationships between risk factors and outcomes, compromising the classification systems’ predictive ability.

Recent technological advances have allowed the development of machine learning (ML) strategies, a branch of artificial intelligence. ML algorithms—which include supervised learning, unsupervised learning, semisupervised learning, and reinforcement learning [13,14]—can use data from several sources, capture complex patterns, and thus may perform better than traditional models [15,16], especially in settings with high variability.

ML has been applied successfully to health care. A systematic review by Kavakiotis and colleagues [17] searched the applications of ML in diabetes research and found that most of the algorithms (85%) used supervised approaches, usually when performing prediction tasks. When it comes to DFU care, a systematic review by Tulloch and colleagues [18] found multiple applications of ML, namely, in classification, image analysis, and segmentation. However, this review focused on identifying the presence and type of DFU but not predicting clinical outcomes in people with DFUs.

Our systematic review aimed to collect all the available evidence assessing the prognostic abilities of ML-based models in predicting clinical outcomes in people with DFUs. We focused on the comparison between models, their performance, and discussed their applicability in the DFU care context. We hope that they can facilitate decision-making and debate the importance of integrating this type of model into daily clinical practice worldwide.

## Methods

This systematic review was conducted using the PRISMA (Preferred Reporting Items for Systematic Reviews and Meta-Analyses) [19] guidelines (Multimedia Appendix 1), and we used the AMSTAR (A Measurement Tool to Assess Systematic Reviews) [20] tool to verify whether the most important aspects have been included in our systematic review. We registered our review in the PROSPERO (International Prospective Register of Systematic Reviews) database in July 2022 and updated in August 2023 under CRD42022308248.

### Search Strategy

We searched the MEDLINE database (PubMed), Scopus, Web of Science, and IEEE Xplore in 2 phases. In the first phase, we performed a search on February 26, 2022, to identify all studies published with no beginning date until December 2021 (inclusive). In the second phase, we updated the search on August 10, 2023, to identify all studies published from January 2022 until July 2023 (inclusive). In both phases, the same queries and databases or registers were used. No restrictions were applied.

Search queries are available in Multimedia Appendix 2. To refine our query, we have used as “satellite” some pertinent papers included in the systematic review by Tulloch and colleagues [18] that addressed a similar topic.

Reference lists of the included papers and previous systematic reviews were reviewed to find additional relevant papers. Experts in the area were contacted to identify any other articles not identified by our query, namely, internal medicine physicians, vascular surgeons, endocrinologists, nurses, podiatrists, and human movement scientists. 

### Inclusion and Exclusion Criteria

Studies were selected based on the PECO-S (Population, Exposure, Comparator, Outcome, and Study type) elements. The criteria applied are the same as those used in the IWGDF systematic review of the classification of foot ulcers in people with diabetes [9] (that would support the development of the IWGDF guidelines), except for those related to the nature of the ML models. These criteria resulted from a consensus decision made by this working group. All the systematic reviews and guidelines produced by the IWGDF followed a standardized methodology [21].

#### Population

Papers were considered eligible if the population included at least 80% of adults with diabetes and a foot ulcer or if a subgroup analysis of such participants was provided. If no subgroup analysis was provided, less than 80% of the sample were people with diabetes, or this information was not provided, the study was excluded.

#### Exposure or Comparator

We defined the exposure of interest as being classified at higher risk (exposure) or lower risk (comparator) by any model developed using artificial intelligence techniques to predict outcomes by assessing more than 1 patient, foot or ulcer characteristic. We also investigated the association between the models’ composing variables (ie, each variable included in the model) and the different outcomes.

#### Outcomes

The authors selected the outcomes for the study using the list provided in the systematic review by Dovell and colleagues [22] as a foundation. Definitions of the outcomes were made according to the document from the IWGD [3]. Our primary outcome was wound healing: reaching intact skin, meaning complete epithelialization without any drainage of a previous foot ulcer site.

As secondary outcomes, we used the following: (1) lower extremity amputation: resection of a segment of a lower limb through a bone or a joint, (2) hospitalization: care in a hospital that requires admission as an inpatient and usually requires an overnight stay, (3) length of stay: period of time in which a person is committed to a hospital, (4) health-related quality of life: a person’s perceived physical and mental health, (5) survival: the state or fact of continuing to live or exist, (6) ulcer-free survival period or time: period of time in which a person is alive and without a foot ulcer, and (7) LEA-free period: period of time in which a person is alive and without a LEA. The paper had to measure at least one of these outcomes to be included in our review.

#### Study Type

We included analytic anterograde longitudinal studies, meaning clinical trials and cohort studies. If a study was presented as an abstract or poster, further searching was done to identify whether it gave origin to a full paper. If not, the study was excluded.

### Eligibility Assessment and Data Extraction

In summary, papers were included if they were anterograde analytical studies that examined the prognostic abilities of ML models in predicting clinical outcomes in a population that included at least 80% of adults with DFU. The search was conducted in 2 phases. In both phases, the studies were reviewed independently by 2 reviewers: MMS and DAR or EJH in the first phase, and MMS and MAS in the second phase. Studies were selected based on their titles and abstracts in the first stage and the complete text of the papers in the second stage. Divergent opinions were resolved by consensus. We used EndNote 20 to manage references and identify duplicates. Subsequently, we used Rayyan QCRI [23] for the blind and independent selection of references to be included in our systematic review. The proportion of agreement between the 2 reviewers was calculated for each stage.

Data were extracted from each included study using a spreadsheet and summarized in tables that included the following information: (1) paper identification (authors, year of publication, and country where study was conducted), (2) methods (study design, inclusion of participants, sample size, follow-up, and context of study), (3) model characteristics (purpose, methods for development, validation conducted, and variable definitions), (4) outcome definition, and (5) results and analysis (participants’ age, type of diabetes, diabetes duration, sex, and measures and statistical methods used). Data were extracted by 1 reviewer (MAS) and confirmed by a second reviewer (MMS). Divergent opinions were solved by consensus.

### Data Synthesis

Due to the expected high level of heterogeneity, meta-analysis was not possible. Thus, we have used the Synthesis Without Meta-Analysis reporting guidelines as a base for our data synthesis [24]. We have grouped the results by the clinical outcome studied. Within each outcome, we ordered studies by the model development stage, study design ( randomized controlled trial, prospective cohort, or retrospective cohort), setting (multicenter or single center), and sample size in our extraction tables, and described the results in a narrative synthesis accordingly. We focused on diagnostic accuracy measures (such as sensitivity, specificity, predictive values, likelihood ratios, and area under the receiver operating characteristic curve [AUROC]) and respective 95% CIs.

### Risk of Bias

The risk of bias was assessed using the Cochrane Risk-of-Bias (RoB 2) tool [25] for randomized controlled trials for impact analysis. If a study had a low risk of bias in all 5 domains, it was classified as at low risk of bias; if some concerns existed in at least 1 domain without any domain with a high risk of bias, it was classified as with some concerns; and if at least 1 domain had a high risk of bias or some concerns existed for multiple domains, it was classified as at high risk of bias.

For observational longitudinal studies of clinical prognosis, both the Quality In Prognosis Studies (QUIPS) tool [26] and the Prediction model Risk Of Bias Assessment Tool (PROBAST) [27] were used. In the case of QUIPS, we evaluated the studies according to 5 of the 6 proposed domains. We considered that the study confounding domain was not pertinent as this paper aims to study the association between variables and outcomes regardless of a causal relationship. Thus, this domain was classified as low risk in all studies. Overall, if a study had a low risk of bias in the 6 domains, it was classified as being at very low risk of bias, in 4-5 domains as being at low risk of bias, and 3 or fewer domains as being at high risk of bias. In the case of PROBAST, if a prediction model had a low risk on all domains relating to bias and applicability, it was classified as at low risk of bias or low concern regarding applicability; if a model had a high risk for at least 1 domain, it should be classified as having high risk of bias or high concern regarding applicability; and if a model had unclear risk in 1 or more domains and had low risk in the remaining domains, it may be classified as having unclear risk of bias or unclear concern regarding applicability. Two reviewers (MMS and MAS) assessed the risk of bias. Divergent opinions were resolved by consensus. The proportion of agreement between the 2 reviewers was calculated.

## Results

### Search Results

In the first phase, we retrieved a total of 1950 references after removing the duplicates. A total of 90 references were selected in the first stage (title and abstract screening), with a proportion of agreement of 95% among the assessors (MMS, DR, and EH). After the second stage (full-text screening), with an agreement of 98%, 4 references were included in our systematic review.

**Figure 1:**
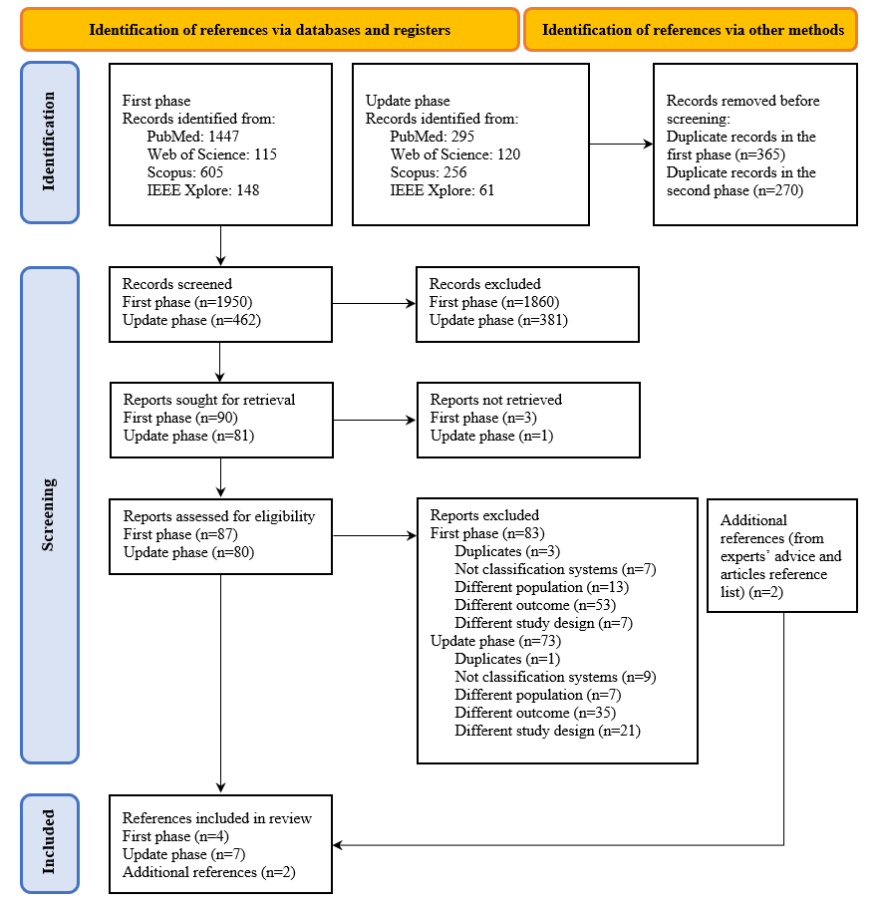
PRISMA flow diagram of the paper selection process.

In the second phase (update of search), we retrieved 462 additional references. A total of 81 references were selected in the first stage, with a proportion of agreement of 90% between the 2 assessors (MMS and MAS). After the second stage, we included 7 additional references in our systematic review, with an agreement of 98%. From searching the references of previous reviews (systematic or not), of included studies, and from contacting experts, we retrieved an additional 2 references. Thus, we included 13 papers reporting on 11 studies (Figure 1). The number of references included differs from the number of included studies, as Wang et al [28,29] and Husers et al [30,31] published 2 studies each using different outcomes but of models developed in the same sample.

### Studies’ Design, Setting, and Population

We have included studies published between 2016 and 2023, conducted in 5 countries: the United States (5/11, 45%) [32-36], China (3/11, 27%) [28,29,37,38], Germany (1/11, 9%) [30,31], Poland (1/11, 9%) [39], and India (1/11, 9%) [40]. Eight studies (8/11, 73%) were retrospective cohorts [28,29,32,34-38,40] and 3 were prospective cohorts [30,31,33,39]. Six studies (6/11, 55%) were single-center [30,31,35,37-40], with sample sizes ranging from 46 to 618 participants (median 201, IQR 126-285), while 5 studies were multicenter [28,29,32-34,36] and had a sample size varying from 204 to 88,898 participants (median 53,354, IQR 362-88,898).

We have separated the results by the clinical outcome (wound healing, LEA, and mortality) and organized the studies included in each table (Multimedia Appendices 3-5) by a higher stage of development (meaning external validation, internal validation, or derivation only), study design with less risk of bias (meaning prospective cohort or retrospective cohort study), multicenter versus single center, and larger sample size. Our search did not retrieve some of the secondary outcomes defined in our protocol, namely, hospitalization, length of stay, health-related quality of life, ulcer-free survival period or time, and LEA-free period.

### Prediction Models’ Characteristics by Clinical Outcome

#### Wound Healing

A total of 5 papers (5/13, 38%) [28,33,35,36,39] used wound healing as an outcome with some variations (Multimedia Appendix 3): 2 studies [33,35] evaluated wound healing, 1 study assessed delayed wound healing [36], 1 study evaluated hard-to-heal wound [28], and 1 study [39] evaluated wound healing failure. Follow-up periods for the mentioned outcomes ranged from 4 to 16 weeks, while 1 study [35] did not explicitly define any period of time for measuring the outcome.

Four studies [28,33,35,39] included only DFUs, with a total of 846 participants. Jung et al [36] included several types of wounds, with 6055 (4% of the overall sample) being neuropathic DFUs and provided a subgroup analysis. The incidence of wound healing varied from 35.1% to 78.8%. Regarding hard-to-heal wounds and wounds with delayed or failed healing, the incidence ranged from 11.6% to 66.0%.

A median of 35 clinical variables per study were assessed for model construction. Final models included between 4 and 865, with a median of 10 variables per study, distributed across 4 categories: demographic characteristics, medical history, laboratory data, and foot-related characteristics (Multimedia Appendix 6). Kim et al [35] also included image-based characteristics retrieved from photographs through the user’s subjective observation and deep learning techniques. The most commonly included were wound area (4/5, 80%), sex (3/5, 60%), and C-reactive protein (3/5, 60%).

Regarding ML methods, 4 studies (4/5, 80%) [28,35,36,39] used multiple techniques simultaneously. The most applied ML method was random forest (RF), which was used in 4 studies (4/5, 80%), followed by support vector machine and least absolute shrinkage and selection operator regression, which was used in 2 studies each (2/5, 40%).

Overall, across the 5 papers, 20 prediction models were created. Participants with missing data were excluded in 2 studies [28,36] and were not reported in 1 study [39]. One study [33] reported the existence of missing values but not the approach to handle them, and 1 study [35] used imputation with a k-nearest neighbors (k-NN) algorithm. As for model validation, every study used internal validation processes. Calibration was evaluated in 2 studies [33,36] using the Hosmer-Lemeshow test and Brier score, and discrimination accuracy was assessed by several measures including the area under the curve (AUC). Reported AUCs in model testing ranged from 0.636 to 0.864. The model that showed apparently better discrimination was developed in the study by Wang and colleagues [28], using 10 clinical variables and the naïve Bayesian classifier. However, 95% CIs were never reported.

#### Lower Extremity Amputation

Eight papers (8/13, 62%) [29-32,34,37,38,40] had LEA as outcome (Multimedia Appendix 4). One paper focused on major LEA [32], another paper on minor LEA [29], and 4 papers assessed simultaneously 2 different types of LEA (minor and major, and major and any) [30,31,34,37]. The remaining papers focused on any form of amputation. The follow-up period for determining LEA occurrence ranged from 6 to 12 months; 4 studies [29,32,37,38] did not mention a predefined follow-up period for determining the outcome.

A total of 417,315 people with diabetes were included. The incidence of major LEA ranged from 5.9% to 12.2%, whereas the incidence of minor LEA ranged from 11.5% to 20.7%. Concerning any form of LEA, the incidence varied from 1.6% to 31.6%.

A median of 21 clinical variables per study were assessed for model construction. Final models included between 7 and 37, with a median of 10 variables per study. Du et al [38] reported only the most relevant variables to model construction, so only those were accounted for. The most frequent variables were age (5/7, 71%), sex, diabetes duration, smoking history, hemoglobin A_1c_, creatinine, albumin, and random blood glucose (all 3/7, 43%) (Multimedia Appendix 7).

Regarding ML techniques, 4 studies (4/7, 57%) used multiple techniques simultaneously [29,32,34,38]. The most used ML method was RF, which was used in 4 studies (4/7, 57%) [29,32,34,38].

Overall, across the 7 papers, 27 prediction models were created. Missing data were inappropriately handled in 2 studies [29,32] and were not reported in the remaining studies. As for model validation, every study conducted internal validation. The exception was the studies by Husers et al [30,31] (which led to 2 papers) that only developed models. Calibration was evaluated in 2 studies [34,37] using the McFadden *R*^2^, isotonic regression, and Brier score, and discrimination accuracy was assessed through AUROC in 5 papers [29-32,37,38]. Reported AUCs ranged from 0.60 to 0.90. The model that showed apparent better discrimination ability was developed in the study by Xie and colleagues [37], using 37 clinical variables and a Light Gradient Boosting Machine. One study reported an accuracy of 94% [40], and 1 study [34] reported only an out-of-bag error rate, which varied from 31% to 63%. Kasbekar et al [40] and Husers et al [30,31] reported 95% CI, allowing comparisons between models.

#### Mortality

Mortality was defined as an outcome in 2 papers (2/13, 15%) [34,38], and, in one of them [34], it was measured after 6 months (Multimedia Appendix 5). A total of 88,944 persons were included. The mortality rate varied from 4.5% to 17.4%.

The final models included a median of 10 variables per study, with Du et al [38] describing only the most relevant variables for model construction. The only repeated variable was age (2/2, 100%) (Multimedia Appendix 8). Both studies used multiple ML techniques including logistic regression and RF in their analysis.

Overall, 8 prediction models were created. Neither of the studies reported missing data. Both studies used processes of internal validation. Calibration was evaluated in 1 study using the McFadden *R*^2^, and discrimination accuracy was assessed, by 1 paper [38], using the AUROC, with reported values ranging from 0.56 to 0.94. The other paper [34] reported only out-of-bag error rate varying from 30% to 68%.

### Risk of Bias

We evaluated the risk of bias according to 2 tools: QUIPS (Multimedia Appendix 9), with a proportion of agreement of 71%, and PROBAST (Multimedia Appendix 10), with a proportion of agreement of 88% for risk of bias and 61% for applicability.

#### Risk of Bias According to QUIPS

All studies presented a high risk of bias and had 2 or 3 (out of the 6 domains) classified as being at low risk (Multimedia Appendix 9). In the study participation domain, all 11 studies were classified as having a moderate or high risk of bias, mainly because most studies did not clearly explain how participants’ sampling was conducted and failed to describe fully eligibility criteria.

In the study attrition domain, 9 studies (9/11, 82%) were classified as having a high risk of bias. Of these, 7 did not mention the proportion of patients who concluded the study [28-32,34,37,38,40], and 2 [35,36], although reported the response rate, did not characterize the excluded patients or the reasons for their exclusion.

As for the prognostic factor measurement domain, all studies were classified as having moderate or high risk of bias. Nine studies (9/11, 82%) [28,29,32,33,35-40] failed to clearly define or explain how to collect all the analyzed variables. Several variables, such as wound area, wound depth, erythema, and adequate arterial flow, are subjective and can lead to different results if measured differently. Two studies [33,36] may have introduced bias due to using several patient centers with no standardized protocols provided. Apart from 1 study [35], in which imputation with a k-NN algorithm was used, missing data were not reported, not addressed, or incorrectly handled.

Concerning the outcome measurement domain, 4 studies (4/11, 36%) were classified as having a moderate or high risk of bias. Three of these studies [33,35,36] failed to define the outcome clearly, and none specified a protocol for outcome assessment. Statistical analysis and reporting were considered in all studies to be at low risk of bias.

#### Risk of Bias According to PROBAST

All studies presented a high risk of bias, and 10 studies presented high or unclear concerns for applicability (Multimedia Appendix 10). Five studies were classified as having high or unclear risk of bias in the participants’ domain due to inadequate or absent eligibility criteria. In the evaluation of applicability, 7 studies had a high or unclear concern for applicability. Similarly, this is mainly due to the inclusion of participants that may differ from the studies’ target population.

In the predictors domain, 9 studies were classified as having unclear risk of bias and unclear risk of applicability. Eight studies [28,29,32,34-38,40] had a retrospective design and failed to mention whether the predictors’ assessment was made without the knowledge of outcome data. Two studies [33,36] included multiple patient centers, and a homogenous assessment of predictors was not clearly guaranteed.

As for the outcomes, 9 papers were classified as having a high or unclear risk of bias. In 4 of these [33,35,36,39], authors failed to report a clear definition for the outcome, compromising its measurement. Another factor that potentially introduced bias was the lack of follow-up reporting (n=5) [29,32,37,38,40]. In the evaluation of applicability, 4 studies had a high concern for applicability due to the lack of clear definitions for the outcomes.

In the analysis domain, all studies were classified as having a high risk of bias. Nine studies had insufficient sample sizes, with only 2 studies [32,34] reaching more than 200 events per variable (EPV) tested—the cutoff considered reasonable to minimize overfitting when using ML techniques. Three studies [28,29,32,36] converted continuous variables into categorical ones, using arbitrary rules for categorization. Regarding missing data, only 1 study [35] addressed missing data correctly, using imputation with a k-NN algorithm. Two studies [28,29,39] failed to avoid selecting variables solely based on univariate analysis, whereas 2 studies [36,40] did not mention how variables were selected. As for model evaluation, 7 studies [28-32,35,38-40] did not report any calibration. The remaining used different methods, including Hosmer-Lemeshow statistics, the Brier score, the McFadden *R*^2^, and the isotonic regression. Discrimination accuracy measures were used by all studies except 1 [34], with AUROC being the most often used. However, uncertainty measures were seldom provided. In terms of validation, apart from 1 (that only derived models) [30,31], every study relied solely on internal validation methods.

## Discussion

### Principal Findings

DFUs have become one of the most important causes of mortality and morbidity in people with diabetes. Guideline-directed foot examinations and treatments and aggressive medical management of diabetes and cardiovascular disease are paramount in improving the prognosis of people with a DFU. However, effective interventions do not work in the same way in all people, and the same management cannot be provided to everyone with diabetes. For that reason, predictive models have been used to stratify people with DFU by their probability of healing, requiring an LEA and dying, so that interventions and resources can be appropriately allocated.

Our systematic review aimed to ascertain whether models using an ML approach and clinical data that are easily accessible in clinical practice could predict clinical outcomes. We have included 11 studies corresponding to 13 papers, mainly from the United States and China (8/11, 73%), retrospective (8/11, 73%), single center (6/11, 55%), and with LEA as the outcome (8/13, 62%).

Other reviews have previously investigated how ML could improve DFU care. For example, a systematic review by Tulloch and colleagues [18] focused on identifying and classifying DFU at a specific moment. In this review, the predictive abilities of ML algorithms were not considered, unlike ours. More recently, a systematic review by Huang and colleagues [41] searched the literature for models that predicted amputation. This study focused on prognosis but considered models that used several methodologies (ML and not ML) and that predicted only 1 outcome (LEA). This review included people with “diabetic foot,” a broader concept than DFU, characterized by ulcers or destruction of the tissues due to infection or peripheral artery disease isolated or both combined.

Although we have included 11 studies (published as 13 papers), we found a total of 55 prediction models focusing on 3 outcomes: healing (5 papers presented 20 models), LEA (7 papers presented 27 models), and mortality (2 papers presented 8 models). Our search did not retrieve some of the outcomes defined in our protocol, such as hospitalization, length of stay, health-related quality of life, ulcer-free survival period or time, and LEA-free period, nor reliability studies, external validation studies, or studies assessing the impact of developing and implementing a predictive model in clinical practice.

For model construction, studies used clinical variables distributed into 4 categories: demographic characteristics, medical history, laboratory data, and foot-related characteristics. The number of variables in the final models varied from 4 to 865.

When it comes to wound healing, variables from the foot-related characteristics group were the most frequently selected, with wound area being used in models from almost all studies. In fact, in previous studies, ischemic ulcers, more extensive and deeper ulcers, plantar ulcers, and ulcers with infection have been associated with poor healing [42,43]. Besides clinical variables, Kim et al [35] also included image-based characteristics from photographs through subjective and deep learning analysis. The subjective observation allowed models to be adequately trained with good performance, turning the utilization of smartphone and tablet photographs for prognosis assessment in clinical practice into a possibility.

Regarding LEA, the most frequently selected variables were age, sex, diabetes duration, smoking history, hemoglobin A_1c_, creatinine, albumin, and random blood glucose. In the case of sex, males have been reported to have higher amputation rates, likely due to behavioral differences [44,45]. As for smoking, studies have suggested its association with LEA by increasing the risk of atherosclerosis and, consequently, of peripheral arterial disease [44,45]. Decreased albumin levels have also been connected to higher LEA rates [45]. As for the remaining variables, a recent systematic review with meta-analysis found no correlation between these and the risk of LEA [45], questioning the methods chosen by these papers to select predictors for model construction. Finally, in the case of mortality, age was the only variable repeated in the 2 selected studies. Despite this being an important outcome, not many studies have sought to create models that could predict mortality.

The most used ML method was RF. It was first described by Breiman [46] and is a supervised method that uses “parallel ensembling,” fitting several decision tree classifiers in parallel, where each tree is trained on a random subset of the training data with replacement (bagging). Majority voting or averages are used to obtain the final result. This method is suitable for both classification and regression problems and has a reduced risk of overfitting, when compared with decision trees. However, it is a time-consuming process that requires more resources and with a more complex interpretation [14].

The most reported discrimination accuracy measure was AUROC, an effective way of summarizing the overall diagnostic accuracy of a model, taking values from 0 to 1. The receiver operating characteristic curve is depicted by using each possible value of a continuous variable as a point with a certain sensitivity and (1–) specificity in discriminating those with and with no clinical condition of interest [47].

There is some variation in the qualitative descriptors of model performance for AUROC thresholds. According to Mandrekar [47], AUROC values of 0.7-0.8 are considered acceptable, 0.8-0.9 are considered excellent, and more than 0.9 are considered outstanding. However, the AUROC is a combined measure of the overall sensitivity and specificity of a model. This implies that 2 models can have identical values, and one performs better in higher sensitivities and the other performs better in higher specificities [48]. Furthermore, AUROC values may overestimate model performance, when the same database is used for both testing and training as happened in most studies. Therefore, interpretation of AUROC values must be done cautiously.

Overall, most models were able to achieve good discrimination ability, with 51% of the reported AUROC values being equal or superior to 0.8. However, all studies were considered to have a high risk of bias, according to QUIPS and PROBAST. First, most studies failed to clearly describe inclusion and exclusion criteria, raising doubt about the potential applicability of final models. Patients lost to follow-up, when reported, were also excluded. Second, most studies did not clearly define the variables considered or describe the methodology adopted to measure them. Some variables, such as those in the foot-related characteristics group, were subjective, and different assessors may measure them in various ways. As a result, without clear definitions and established protocols for variables’ measurement, predictive models’ validity and application to clinical practice may be compromised. This lack of standardization also applies to outcomes, where clear definitions are essential to guarantee consistency. In the case of the studies considering wound healing as an outcome, different variations were used (delayed wound healing, hard-to-heal wound, and wound healing failure). However, most did not provide a definition or a methodology for outcome measurement. Also, follow-up periods varied widely among studies, which makes comparing predictive models even more challenging. These variations and lack of standardization were expected; thus, no meta-analysis was considered from inception. Third, there were some problems in the analysis domain. Studies had insufficient sample sizes, with only 2 reaching more than 200 EPV [32,34]. “EPV” refers to the number of events (ie, number of patients in which the outcome of interest has occurred) relative to the number of regression coefficients used (ie, number of variables considered for model development) [27]. In ML-based models, higher EPV (often more than 200) are needed to minimize overfitting [49]. Also, most studies used the same sample set to develop (train) and validate (test) the models, which can increase the overestimation of the accuracy due to overfitting. Missing data were not reported, not addressed, or incorrectly handled, except in 1 study that used imputation methods.

Although reported models showed promise in predicting clinical outcomes, most are not available for immediate applicability to clinical practice. Only 3 studies presented web-based interactive models: 2 [29,32] for LEA prediction and 1 [28] for hard-to-heal DFU prediction. In addition, several studies developed and internally validated several models using different methods in the same sample without providing all required accuracy measures (focusing on AUROC) and seldomly reporting 95% CI and calibration measures—making it impossible to compare and select specific models to be externally validated before they can be used in clinical practice. The lack of reporting of calibration measures is a common issue in predictive modeling research that can lead to incorrect and potentially harmful clinical decisions, especially for models that focus on estimating the likelihood of a clinical event for each individual.

When validating (internally or externally) predictive models, it is imperative to evaluate calibration using appropriate measures in addition to visualization strategies (such as the calibration curve). The latter helps us understand how the algorithm performs in a particular setting, where predictions may go wrong (over or underestimating the probability of a clinical event), and whether the algorithm can benefit from updating [50].

The lack of 95% CI impairs the comparison of the several models developed within each study to understand the measure with the highest value of each model, the comparison between models derived by different authors, and the comparison between validating studies. We should require authors writing on this topic to report each measure with the corresponding 95% CI or similar measures of uncertainty.

Due to the high risk of bias of all the included studies and the high discrepancy in many aspects, we could not select criteria to prioritize results for summary and synthesis besides the ordering shown in our tables. Also, due to the lack of sufficient studies using the same outcome (with identical definitions), we consider that there is high heterogeneity without formal ascertainment and indirectness of the results.

Another aspect we would like to emphasize is that applying ML models in clinical environments presents several practical challenges. These models must be integrated into clinical and digital workflows to reach their full potential. However, specific clinician-related barriers may arise. For instance, clinicians may find it difficult to trust these systems as it can be challenging to understand how they work, especially in nonexplainable models. Additionally, some clinicians may view this new technology as threatening their professional autonomy and be reluctant to adopt it. User-unfriendly, time-consuming data collection and poorly designed interfaces may lead to non or incomplete adoption or early abandonment. From a legal perspective, using complex decision support systems raises essential concerns. In medical negligence cases, for example, it may be challenging to determine which entity holds liability—the clinician or the algorithm. As artificial intelligence becomes part of the standard of care, the roles of health care professionals will evolve, requiring them to develop new skills [51].

ML models are developed using considerable data, and data quality will directly impact model performance. It is important to note that these methods can amplify implicit bias and discrimination if trained on data that reflect the health care disparities experienced by groups defined by race, ethnicity, gender, sexual orientation, socioeconomic status, or geographic location. To mitigate these pitfalls, algorithms must be trained on fair datasets that include and accurately represent social, environmental, and economic factors that influence health [51].

On the other hand, we believe that ML models can provide an individual prognostic estimation for people with DFU. It is evident in the IWGDF guidelines [10], based on a systematic review focusing on traditional methods [9], that none of the classification systems available can adequately fit this role. A model should focus on calibration measures that depend on more included variables for individual prognostication. In contrast, for the remaining purposes (communication between professionals, characterization of complex cases, and audit), the models should focus on discrimination measures that diminish with the inclusion of more variables. Also, operationally, they should have a minimal amount of variables that can better explain variability between groups of people with similar probability of developing the outcome.

For all this, we believe that to make it possible to provide “the right treatment, to the right person at the right time” [52], more focus on ML models research should be provided so that they can be implemented safely in clinical practice.

### Limitations

Our systematic review presents some limitations. First, we conducted our search in 2 phases, involving different reviewers in the selection process, which may have introduced some variability. However, the agreement proportion was high and comparable when comparing both phases. Second, a systematic review is only as good as the included studies. Due to the high risk of bias and the high heterogeneity of included studies (mainly due to a lack of uniform variable definitions, outcome definitions, and follow-up periods), we have decided that a meta-analysis should not be performed. However, we would like to highlight that this reality is not as different from the results achieved in our systematic review of “classic” classification systems. Finally, the complexity of the ML methodology prevented further explanation of the studied models, which may lead to some distrust from health care providers when considering the application of such models in clinical practice.

### Future Research

Future research should consider some of the issues we identified in the included studies. Inclusion and exclusion criteria should be listed and defined, and only variables available at the point of inclusion in the study should be used. Excluding participants due to a lack of complete follow-up or missing data indicates that the authors have conducted a “complete case analysis.” A complete case analysis means that only participants with data available for all the included variables and with the minimum follow are included in the dataset. This approach is not recommended due to potential selection bias, as an absence of information about specific variables can have clinical meaning. In addition, if the authors do not report the number of participants excluded due to these criteria, it is impossible to ascertain this methodology’s impact.

Authors should clearly define model variables and the methodology used to measure them objectively and reproducibly. This reporting detail is particularly important when dealing with subjective variables and when multiple centers and assessors are involved. Conducting prospective studies with a standardized protocol to collect variables is always recommended in these circumstances. In addition, when developing DFU prediction models, it is recommended that the variables to be tested and included in the model should be selected according to clinical sense and available evidence provided by published studies and not merely by statistical significance.

When selecting and defining clinical outcomes, we recommend that those with well-documented definitions in the literature be preferred [3] and from previously defined core outcome sets [53]. For example, “wound healing” should be preferred over some of its variations (delayed wound healing, hard-to-heal wound, and wound healing failure) so that future comparisons among different studies are possible. Additionally, other outcomes, such as quality of life, which are clinically relevant, should be further used.

Another major issue we identified was the inadequate sample sizes of many studies in order to develop models with many included variables. Authors should select the number of variables to be tested by considering the number of events in their dataset and not just the availability of information.

Models undergoing external validation are lacking. Thus, future studies should validate their models using data external to the development sample, using participants from different centers and settings. To ensure that this step is possible, the model needs to be translated into other settings. We encourage authors to conduct external validation studies using some of the models presented in our systematic review. However, without consistent reporting of discrimination and calibration measures, it would be difficult to determine whether such models perform adequately.

### Conclusions

This systematic review found several ML-based models that could predict clinical outcomes with good discrimination ability in people with DFU, showing promising results. However, studies presented a high risk of bias with several applicability issues that compromise the ready applicability of such models in clinical practice. Further studies with stricter methodology are needed so that patients who have diabetes and a foot ulcer can benefit from the recent advancements of artificial intelligence applied to health care.

## Appendix

Multimedia Appendix 1 PRISMA checklist.

Multimedia Appendix 2 Search queries on PubMed, Web of Science, Scopus, and IEEE Xplore.

Multimedia Appendix 3 Characteristics of the included studies organized by model development stage, study design, setting, and sample size: wound healing as outcome.

Multimedia Appendix 4 Characteristics of the included studies organized by model development stage, study design, setting, and sample size: lower extremity amputation as outcome.

Multimedia Appendix 5 Characteristics of the included studies organized by model development stage, study design, setting, and sample size: mortality as outcome.

Multimedia Appendix 6 Distribution of clinical variables included in the final models by categories having healing as outcome.

Multimedia Appendix 7 Distribution of clinical variables included in the final models by categories having lower extremity amputation as outcome.

Multimedia Appendix 8 Distribution of clinical variables included in the final models by categories having mortality as outcome.

Multimedia Appendix 9 Quality In Prognosis Studies (QUIPS) tool results of included studies.

Multimedia Appendix 10 Prediction model Risk Of Bias ASsessment Tool (PROBAST) tool results of included studies.

## Abbreviations

AMSTAR: A Measurement Tool to Assess Systematic Reviews
AUC: area under the curve
AUROC: area under the receiver operating characteristic curve
DFU: diabetes-related foot ulceration
EPV: events per variable
IWGDF: International Working Group on the Diabetic Foot
k-NN: k-nearest neighbor
LEA: lower extremity amputation
ML: machine learning
PECO-S: Population, Exposure, Comparator, Outcome, and Study type
PRISMA: Preferred Reporting Items for Systematic Reviews and Meta-Analyses
PROBAST: Prediction model Risk Of Bias Assessment Tool
PROSPERO: International Prospective Register of Systematic Reviews
QUIPS: Quality In Prognosis Studies
RF: random forest
