# Supplementary material for: Diabetic Foot Ulcer Classification Models Using Artificial Intelligence and Machine Learning Techniques: Systematic Review
Source: J Med Internet Res. 2025 Sep 24;27:e69408. doi: 10.2196/69408 (PMC12508669; doi:10.2196/69408)
Supplement: Multimedia Appendix 2 [file jmir_v27i1e69408_app2.doc]

**Multimedia Appendix 2.** Search queries on PubMed, Web of Science, SCOPUS and IEEE Xplore.

| PubMed | ("deep learning" [MeSH Terms] OR "deep learning" [All Fields] OR "artificial intelligence" [MeSH Terms] OR "artificial intelligence" [All Fields] OR "deep learning" [MeSH Terms] OR "deep learning" [All Fields] OR "random forest" [All Fields] OR "clinical decision-making"[MeSH Terms] OR "clinical decision-making"[All Fields] OR "medical decision-making"[All Fields] OR "decision support systems, clinical"[MeSH Terms] OR "decision support systems clinical"[All Fields] OR "clinical decision support systems"[All Fields] OR "decision support techniques"[MeSH Terms] OR "decision support techniques"[All Fields] OR "machine learning"[MeSH Terms] OR "machine learning"[All Fields] OR "data mining"[MeSH Terms] OR "data mining"[All Fields] OR "neural networks, computer"[MeSH Terms] OR "neural networks computer"[All Fields] OR "computer neural network*"[All Fields] OR "neural network*"[All Fields] OR ("phenotype"[MeSH Terms] OR "phenotype"[All Fields] OR "phenotypes"[All Fields] OR "phenotyped"[All Fields] OR "phenotypic"[All Fields] OR "phenotypical"[All Fields] OR "phenotypically"[All Fields] OR "phenotyping"[All Fields] OR "phenotypings"[All Fields]) OR "convolutional"[All Fields] OR automat* [Title/Abstract]) AND ("Diabetic Foot"[MeSH Terms] OR ("diabete"[All Fields] OR "diabetes mellitus"[MeSH Terms] OR ("diabetes"[All Fields] AND "mellitus"[All Fields]) OR "diabetes mellitus"[All Fields] OR "diabetes"[All Fields] OR "diabetic"[All Fields] OR "diabetics"[All Fields] OR "diabets"[All Fields]) AND ("ulcer"[MeSH Terms] OR "ulcer"[All Fields] OR "ulcerate"[All Fields] OR "ulcerated"[All Fields] OR "ulcerates"[All Fields] OR "ulcerating"[All Fields] OR "ulceration"[All Fields] OR "ulcerations"[All Fields] OR "ulcerative"[All Fields] OR "ulcers"[All Fields] OR "ulcerous"[All Fields]) OR ("amputate"[All Fields] OR "amputated"[All Fields] OR "amputating"[All Fields] OR "amputation"[MeSH Terms] OR "amputation"[All Fields] OR "amputations"[All Fields] OR "amputed"[All Fields])) |
| --- | --- |
| Web of Science | (TI= "deep learning" OR AB="deep learning" OR TI="artificial intelligence" OR AB="artificial intelligence" OR TI= "random forest" OR AB="random forest" OR TI="decision making" OR AB="decision making" OR TI="decision support" OR AB="decision support" OR TI="machine learning" OR AB="machine learning" OR TI="data mining" OR AB="data mining" OR TI="neural network" OR AB="neural network" OR TI="phenotype" OR AB="phenotype" OR TI="convolutional" OR AB="convolutional") AND ((TI="foot" OR AB="foot") AND (TI="ulcer" OR AB="ulcer")) |
| SCOPUS | ( TITLE-ABS-KEY ( deep AND learning ) OR TITLE-ABS-KEY ( artificial AND intelligence ) OR TITLE-ABS-KEY ( random AND forest ) OR TITLE-ABS-KEY ( decision AND making ) OR TITLE-ABS-KEY ( decision AND support ) OR TITLE-ABS-KEY ( machine AND learning ) OR TITLE-ABS-KEY ( data AND mining ) OR TITLE-ABS-KEY ( neural AND network ) OR TITLE-ABS-KEY ( phenotype ) OR TITLE-ABS-KEY ( convolutional ) ) AND (( TITLE-ABS-KEY ( foot ) AND TITLE-ABS-KEY ( ulcer ) ) |
| IEEE Xplore | ("All Metadata":diabetic foot ulcer) AND ("All Metadata":classification)  ("All Metadata":wound) AND ("All Metadata":chronic) AND ("All Metadata":classification)  ("All Metadata":diabetic foot) AND ("All Metadata":healing)  ("All Metadata":diabetic foot) AND ("All Metadata":convolutional)  ("All Metadata":diabetic foot) AND ("All Metadata":amputation) |
