# Supplementary material for: Diabetic Foot Ulcer Classification Models Using Artificial Intelligence and Machine Learning Techniques: Systematic Review
Source: J Med Internet Res. 2025 Sep 24;27:e69408. doi: 10.2196/69408 (PMC12508669; doi:10.2196/69408)
Supplement: Multimedia Appendix 3 [file jmir_v27i1e69408_app3.doc]

**Multimedia Appendix 3.** Characteristics of the included studies organized by model development stage, study design, setting and sample size: wound healing as outcome.

| **Reference** | **Study design, setting and follow-up** | **Study population and characteristics** | **Development/ validation** | **Variables assessed** | **Primary and secondary outcomes** | **Results** | **Comments** |
| --- | --- | --- | --- | --- | --- | --- | --- |
| Margolis et al, 2022 [33] | Prospective cohort  Multicenter  4 wound care centers in USA  Inclusion period: NR  Follow-up period: 16 weeks | n = 204 people with plantar DFU  n=1 (0.5%) lost to follow-up  n=2 (1.0%) insufficient follow up  Mean age: 58 years  73% male  Mean diabetes duration: 18 years  Inclusion criteria: adult-onset DM; DFU on the plantar aspect of the foot eligible for standard care; adequate arterial flow for healing; ≥ 40 years at the time of DFU diagnosis | Development (train):  LASSO regression model  Calibration:  Hosmer-Lemeshow statistics  Internal validation (test):  10-fold cross-validation | 26 variables assessed; 4 variables included in the model  wound duration, wound area, BMI, adequate arterial flow | Wound healing at 16 weeks | 35.1% healed  AUC: 0.7212 | Inclusion period NR  Missing values reported, but approach NR  Information provided does not allow model application  95% CI not reported  No external validation |
| Poradzka and Czupryniak, 2023 [39] | Prospective cohort  Single center  University hospital in Poland  Inclusion period: February 2018 to March 2019  Follow-up period: 3 months | n = 164 consecutive people admitted with DFU (with 199 feet with DFU)  Mean age: 61 years  75% male  Mean diabetes duration: 17 years  Inclusion criteria: people admitted with at least one DFU (according to IWGDF definitions)  Exclusion of n=13 (7.3%)  2 did not agree to take part in study  2 withdrew consent  2 lost to follow-up  7 died during the follow-up | Development (train):  Artificial neural network (70% for teaching and 15% for validating of sample randomly selected)  Logistic regression  Internal validation (test): 15% of sample randomly selected | 35 variables tested in univariate or multivariate analysis, reducing the group to 6 (based on significance): probe-to-bone test, ESR, CRP, foot skin temperature, DFU area, DFU duration  Added to the model (based on previous study):  prior amputation, presence of blood flow in the Doppler probe, ABI | Healing failure (versus complete healing with or without LEA) | 66% of ulcers without complete healing  ANN  AUC: 0.85  Accuracy: 82.9%  Sensitivity: 91.6%  Specificity: 66.2%  PPV: 84.5%  NPV: 80.4%  F1 score: 87.9%  Logistic regression  Teaching  AUC 0.73  Testing  AUC 0.67 | Single center study  Analysis by limb and not person  n=4 healed after minor LEA and were included in the healed success group  No missing values reported  Excluded lost to follow-up  Logistic regression model not provided  No external validation  95% CI not reported  No calibration measures reported |
| Jung et al, 2016 [36] | Retrospective cohort  Multicenter  68 Healogics wound care centers in USA  Inclusion period: 2009 to 2013  Follow-up period: mean 52 days | n= 6055 neuropathic DFUs  (4% of overall sample)  Mean age: 66 years  53% male  Mean diabetes duration: NR | Development (train):  80% of overall sample  LASSO regression; random forest; gradient boosted tree  Calibration:  Brier reliability of 0.00018  Internal validation (test)  20% of overall sample | 865 predictors which sum up in 52 main variables:  centre code, gender, age, palliative care, complex care, area*, length*, width*, depth, grade, thickness, temperature, exudate, margin, epithelization, tunnelling, undermining, exposed bone, exposed joint, exposed muscle, exposed tendon, brawny induration, oedema, excoriation, callus, crepitus, fluctuance, friable, rash, atrophy blanche, cyanosis, ecchymosis, erythema, hemosiderosis, pallor, rubor, tenderness on palpation, slough, delayed recurrence, result of accident, wound recurrence, clustered wound, pending amputation on presentation, granulation quality, granulation amount, primary insurance, number of wounds, duration of care, previous wound count, ICD9 codes, wound type,  wound location  (all variables were collected at onset of care; variables with asterisks were collected at onset of care and 1 week later) | Delayed wound healing (≥15 weeks to closure) | 11.6% delayed wound healing  Best was gradient boosted tree (only one reported)  AUC: 0.823 | Retrospective  Follow-up period not clear  Patients lost to follow-up were excluded  Only 14% of wounds DFUs and only for neuropathic DFUs (4%) exists subgroup analysis without stratification in random split sample  Not clear of number of people with neuropathic DFU  Calibration measures only for overall sample  Outliers and missing values were excluded  No external validation  95% CI not reported |
| Wang et al, 2022a [28] | Retrospective cohort  Multicenter  2 tertiary hospitals in eastern China  Inclusion period: January 2018 to December 2019  Follow-up period: NR | n = 362 people with DFU  > 63 years  71% male  >12 years duration  Inclusion criteria: 18–89 years, University of Texas Grade 3 DFU  Exclusion criteria: tumour-induced ulcers; previous major LEA; abandoned treatment; had incomplete information | Development (train):  70% of sample randomly selected  6 different models  General linear regression  Naïve Bayesian  Support vector machine  Random forest  k-nearest neighbour  Adaptive boosting  Internal validation (test):  30% of sample randomly selected | 21 variables assessed  10 variables selected:  6 selected after univariate regression analysis (sex,  random blood glucose, diabetic retinopathy, peripheral arterial disease, smoking history, and CRP); 4 selected based on literature and experience (insulin use, wound area, serum albumin, and serum creatinine) | Hard-to-heal DFU (PAR < 50% by week 4) | 54% hard-to-heal DFU  Adaptive boosting  Accuracy: 0.713  PPV: 0.63  Sensitivity: 0.674  F1 score: 0.667  AUC: 0.804  General linear regression  Accuracy: 0.750  PPV: 0.654  Sensitivity: 0.791  F1 score: 0.68  AUC: 0.826  K-nearest neighbor  Accuracy: 0.713  PPV: 0.611  Sensitivity: 0.767  F1 score: 0.688  AUC: 0.817  Naïve Bayesian (model [https://predicthardtoheal.azurewebsites.net](https://predicthardtoheal.azurewebsites.net/))  Accuracy: 0.750  PPV: 0.629  Sensitivity: 0.907  F1 score: 0.744  AUC: 0.864  Random forest  Accuracy: 0.722  PPV: 0.614  Sensitivity: 0.814  F1 score: 0.66  AUC: 0.827  Support vector machine  Accuracy: 0.759  PPV: 0.660  Sensitivity: 0.814  F1 score: 0.694  AUC: 0.819 | Retrospective  Those lost to follow-up and with missing values were excluded  95% CI not reported  No calibration measures reported  No external validation |
| Kim et al, 2020 [35] | Retrospective cohort  Single-center  Podiatry and wound clinic USA  Inclusion period: Nov 2014 to Jul 2017  Follow-up period: NR | n = 113 people with DFU (208 DFU)  Mean age: >58.5 years  % male not clear  Mean diabetes duration: NR  Exclusion criteria: previously infected DFU resulting in complete epithelization; previously DFU marked as healed that re-ulcerated at same location; DFU without image at initial visit  42 people (173 DFU) excluded | Development (train):  75% of sample  random forest and support vector machine  Internal validation (test):  25% of sample  3-fold cross-validation and grid-search | 2133 Image features (DFU manually segmented from photographs): 85 hand-crafted image features (colour and texture) plus 2048 deep learning-based features (extracted from the GAP layer of ResNet50)  48 clinical variables:  wound length, width, depth; foot, age, sex, ethnicity, CCI, diabetic retinopathy, lymphocyte count, HbA1c, albumin, pre-albumin, CKD stage coded, CRP, ESR, infection, probe-to-bone test, X-ray, MRI performed, Tc99 performed, dorsalis pedis pulse measured, posterior tibial pulse measured, ankle systolic pressure measured, toe systolic pressure measured, TcPO_2_, BSA, BMI, TCC, offload, immunosuppressants, oral steroids, antihypertensives, oral hypoglycemics, canagliflozin, insulin, heparin, allopurinol, NSAIDs, aspirin, warfarin, Xa inhibitors, race, University of Texas stage, grade | Healing (healed or not healed) | 78.8% of DFU healed  Models built with all available features  Development  Random forest  AUC: 0.734  Accuracy: 0.811  PPV: 0.828  Sensitivity: 0.923  F1: 0.873  Support vector machine  AUC: 0.734  Accuracy: 0.811  PPV: 0.828  Sensitivity: 0.923  F1: 0.873  Validation  Random forest  AUC: 0.691  Accuracy: 0.671  PPV: 0.743  Sensitivity: 0.823  F1: 0.777  Support vector machine  AUC: 0.735  Accuracy: 0.726  PPV: 0.780  Sensitivity: 0.854  F1: 0.814  Models built only with image features  Development  Random forest  AUC: 0.760  Accuracy: 0.811  PPV: 0.852  Sensitivity: 0.885  F1: 0.868  Support vector machine  AUC: 0.794  Accuracy: 0.784  PPV: 0.909  Sensitivity: 0.769  F1: 0.833  Validation  Random forest  AUC: 0.683  Accuracy: 0.646  PPV: 0.795  Sensitivity: 0.671  F1: 0.727  Support vector machine  AUC: 0.691  Accuracy: 0.561  PPV: 0.840  Sensitivity: 0.471  F1: 0.600  Models built only with clinical features  Development  Random forest  AUC: 0.636  Accuracy: 0.784  PPV: 0.765  Sensitivity: 1.000  F1: 0.867  Support vector machine  AUC: 0.657  Accuracy: 0.703  PPV: 0.800  Sensitivity: 0.769  F1: 0.784  Validation  Random forest  AUC: 0.693  Accuracy: 0.707  PPV: 0.757  Sensitivity: 0.875  F1: 0.805  Support vector machine  AUC: 0.701  Accuracy: 0.591  PPV: 0.810  Sensitivity: 0.567  F1: 0.652  Models built only with deep learning features  Development  Random forest  AUC: 0.670  Accuracy: 0.757  PPV: 0.793  Sensitivity: 0.885  F1: 0.836  Support vector machine  AUC: 0.670  Accuracy: 0.757  PPV: 0.793  Sensitivity: 0.885  F1: 0.836  Validation  Random forest  AUC: 0.692  Accuracy: 0.720  PPV: 0.764  Sensitivity: 0.880  F1: 0.815  Support vector machine  AUC: 0.726  Accuracy: 0.707  PPV: 0.737  Sensitivity: 0.912  F1: 0.813  Random forest + support vector machine models with imaging features alone outperformed models trained with deep learning-based features alone (*p* = 0.013)  Support vector machine models trained with imaging features alone outperformed models with clinical features alone (*p* = 0.031) | Retrospective  Single-center study  Patient follow-up period NR  High rate of patient exclusion  Those lost to follow-up were excluded  Not described how sample was split for train and test  95% CI not reported  Correspondence between people and DFU not explained or if analysis by DFU  No calibration measures reported  No external validation |

ABI: ankle-brachial index; ANN: artificial neural network; positive predictive value; AUC: area under the curve; BMI: body mass index; BSA: body surface area; CCI: Charlson comorbidity index; CI: confidence interval; CKD: chronic kidney disease; CRP: C-reactive protein; DFU: diabetic foot ulcer; DM: diabetes mellitus; ESR: erythrocyte sedimentation rate; GAP: global average pooling; ICD-9: International Classification of Diseases, 9th Revision; IWGDF: International Working Group on the Diabetic Foot; LASSO: least absolute shrinkage and selection operator; LEA: lower extremity amputation; MRI: magnetic resonance imaging; NPV: negative predictive value; NR: not reported; NSAIDs: non-steroidal anti-inflammatory drugs; PAR: percent area reduction; ResNet50: residual network 50; Tc99: technetium-99 bone scan; TCC: total contact cast use; TCPO_2_: transcutaneous oxygen pressure; USA: United States of America.
