# Supplementary material for: Diabetic Foot Ulcer Classification Models Using Artificial Intelligence and Machine Learning Techniques: Systematic Review
Source: J Med Internet Res. 2025 Sep 24;27:e69408. doi: 10.2196/69408 (PMC12508669; doi:10.2196/69408)
Supplement: Multimedia Appendix 4 [file jmir_v27i1e69408_app4.doc]

**Multimedia Appendix 4.** Characteristics of the included studies organized by model development stage, study design, setting and sample size: lower extremity amputation as outcome.

| **Reference** | **Study design, setting and follow-up** | **Study population and characteristics** | **Development/ validation** | **Variables assessed** | **Primary and secondary outcomes** | **Results** | **Comments** |
| --- | --- | --- | --- | --- | --- | --- | --- |
| Stefanopoulos et al, 2024 [32] | Retrospective cohort  Multicenter  National Inpatient Sample database from community hospitals in USA  Inclusion period: 2008 to 2014  Follow-up period  Mean LoS of 7.3 (±7.5) days on no LEA group and 12.9 (±10.5) days on LEA group | n = 326,853 people admitted with DFU  Mean age: >62 years 36% male  Mean diabetes duration: NR  Exclusion criteria: NR | Development (train):  CTREE with LASSO regression + random forest analysis  70% of sample randomly selected  Internal validation (test):  30% of sample randomly selected + boosting (10 unique 5x boosted data sets) | 39 variables tested, only 19 with OR provided:  Gangrene, septic shock, peripheral vascular disease, weight loss, septicemia, systematic infection, anemia, age ≥ 40, bacteremia, elective procedure, osteomyelitis, leukocytosis, paralysis, renal failure, depression, male, congestive heart failure, hypertension, urinary tract infection, smoking  Top 5 contributing variables (p<0.001):  gangrene, osteomyelitis, systemic infection, peripheral vascular disease and weight loss | Major LEA | 5.9% major LEA  5- and 10- variables model  Unboosted and boosted  Train  Accuracy 78 %  Sensitivity 76 %  Specificity 79 %  Test  Accuracy 78 %  Sensitivity 76 %  Specificity 79 %  AUC 0.84  Random forest  Train and boosted  Accuracy 78 %  Sensitivity 76 %  Specificity 80 %  Boosted  AUC 0.83 | Retrospective  Patient follow-up period NR  Those with missing values or died during follow-up were excluded  Only included risk factors with ICD-9 code entry  95% CI mostly not reported  No external validation  No calibration measures reported  Final interactive model available on: https://grenut.shinyapps.io/amputation/ |
| Austin et al, 2022 [34] | Retrospective cohort  Multicenter  Fee-for-service Medicare database, USA  Inclusion period: 2015  Follow-up period: 6 months | n = 88,898 people with DFU and PAD  Mean age: 77 years  53 % female  Mean diabetes duration: NR  Inclusion criteria: newly diagnosed with concomitant PAD and diabetes; USA residents; age between 65 and 95; Medicare beneficiaries for 1 year following index date (first claim containing first diabetes-related ICD-9 or 10 code); ulcer diagnosis during first 6 months of the index year; outcome-free (alive, no reinterventions or amputations) for at least 6 months after index date | Development (train):  2/3 of sample  Logistic regression; random forest, using a data set randomly divided into training (2/3) and testing (1/3) groups  Calibration of logistic regression model: McFadden R^2^  Internal validation (test)  1/3 of sample | 9 variables tested  Charlson comorbidity index, sex, race, age at diagnosis, Medicare-Medicaid dual-eligibility status, urban/rural indicator, HbA1c, foot exam, vascular imaging study | LEA (both minor and major) | LEA rate was 1.6%  Logistic regression  McFadden R^2^:  LEA: 0.071  Out-of-bag error rate:  LEA: 63%  Random forest  Out-of-bag error rate:  LEA: 31% | Retrospective  Insurance based database  No missing values reported  Excluded lost to follow-up  Information provided does not allow model application  Not described how sample was split for train and test  No external validation  Calibration measures reported only for logistic regression model  95% CI not reported |
| Wang et al, 2022b [29] | Retrospective cohort  Multicenter  2 tertiary hospitals in eastern China  Inclusion period: January 2018 to December 2019  Follow-up period: NR | n = 362 people with DFU  > 63 years  71% male  >12 years duration  Inclusion criteria: 18–89 years, University of Texas Grade 3 DFU  Exclusion criteria: tumour-induced ulcers; previous major LEA; abandoned treatment; had incomplete information | Development (train):  70% of sample randomly selected  5 different models  (logistic regression; random forest; decision tree; support vector machine; and XGBoost), using a data set randomly divided into training (70%) and testing (30%) groups; SMOTE method was used to solve the imbalance between non-LEA and minor LEA groups  Internal validation (test):  30% of sample randomly selected; 10-fold cross-validation | 21 variables assessed  9 variables selected by univariate analysis  random blood glucose, years of diabetes, cardiovascular disease, peripheral arterial disease, smoking history, albumin, creatinine, CRP and DFU history | Minor LEA | 20.7% minor LEA  For minor LEA (after SMOTE):  Decision tree  Accuracy: 0.744  PPV: 0.828  Sensitivity: 0.616  F1 score: 0.707  AUC: 0.813  Random forest  Accuracy: 0.797  PPV: 0.823  Sensitivity: 0.756  F1 score: 0.788  AUC: 0.857  Logistic regression  Accuracy: 0.640  PPV: 0.640  Sensitivity: 0.640  F1 score: 0.640  AUC: 0.739  Support vector machine  Accuracy: 0.663  PPV: 0.689  Sensitivity: 0.593  F1 score: 0.638  AUC: 0.767  XGBoost (model available at https://dfuprediction.azurewebsites.net/)  Accuracy: 0.814  PPV: 0.846  Sensitivity: 0.767  F1 score: 0.805  AUC: 0.881 | Retrospective  Those lost to follow-up and with missing values were excluded  95% CI not reported  No calibration measures reported  No external validation |
| Xie et al, 2022 [37] | Retrospective cohort  Single-center  University Hospital in China  Inclusion period: 2009 to 2020  Follow-up period: NR | n = 618 people admitted with DFU  Mean age: 66 years  62% male  Mean diabetes duration: 10 years  Exclusion criteria: <18 years; patients  who died before hospital discharge; patients who underwent LEA before being referred to the hospital; patients who were referred to other medical institutions during the treatment period | Development (train):  LightGBM  60% of sample randomly selected  Calibration:  Isotonic regression + Brier score  Internal validation (test):  5-fold cross validation  20% of sample randomly selected  SHAP algorithm for model explanation | 37 variables included in the model:  Age, sex, body mass index, diabetes duration, smoking history, pre-hospital delay, hypertension, coronary heart disease, heart failure, cerebral infarction, diabetic neuropathy, diabetic retinopathy, diabetic nephropathy, peripheral vascular disease, arterial occlusion, gangrene, prior DFU, prior amputation, HbA1C, random blood glucose, white blood cell, percentage of neutrophils, hemoglobin, serum potassium, serum sodium, serum creatinine, serum albumin, total cholesterol, triglyceride, LDL-C, HDL-C, antihyperglycemic drugs, insulin use, Wagner classification system, WIfI classification system (wound; ischemia; foot infection) | Minor LEA and major LEA | 11.5% minor LEA, 7.6% major LEA  Overall:  AUC: 0.90  Sensitivity: 87.1%  Specificity: 74.4%  NPV: 79.7%  PPV 86.3%  Brier score: 0.086  Non-LEA:  AUC: 0.90  Sensitivity: 95.0%  Specificity: 69.6%  NPV: 76.2%  PPV 93.2%  Minor LEA:  AUC: 0.86  Sensitivity: 64.3%  Specificity: 94.5%  NPV: 95.4%  PPV 60.0%  Major LEA:  AUC: 0.85  Sensitivity: 33.3%  Specificity: 97.3%  NPV: 94.9%  PPV 50.0% | Retrospective  Single-center study  Patient follow-up period NR  No missing values reported  Information provided does not allow model application  95% CI not reported  No external validation |
| Kasbekar et al, 2017 [40] | Retrospective cohort  Single-center  Hospital in India  Inclusion period: Jun 2011 to Jun 2013  Follow-up period: 1 year | n = 301 people admitted due to DFU  Mean age: NR  Male: NR  Mean diabetes duration: NR  Inclusion criteria: lower extremity ulcer, diabetes  Exclusion criteria: those  died, or lost to follow-up | Development (train):  n=250  C5.0 (decision tree algorithm)  Internal validation (test):  n=51 | 21 variables:  diabetes treatment, duration of leg symptoms, treatment for leg symptoms, history of regular smoking, history of regular alcohol intake, other comorbidities, age, sex, weight, Wagner classification, blood haemoglobin, serum creatinine, blood prothrombin time, serum albumin, serum bilirubin, random blood glucose level, HbA1C levels, x-ray of the affected foot, pus culture and sensitivity examination of the wound, flow through arterial Doppler, length of stay | LEA | 27.6% LEA  Single C5.0 Tree  Decision tree  If absent or monophasic peripheral flow → high risk of LEA  If normal, triphasic, or biphasic grade DFU with Wagner  Those with grade 4 and 5 → high risk of LEA  Development  Accuracy: 96%  Validation  Accuracy: 94%  Boosted C5.0 Tree Ensemble  (using 17 variables)  Accuracy: 96%  (95% CI: 0.865 – 0.995) | Retrospective  Single-center study  Baseline characteristics NR  No missing values reported  Those lost to follow-up were excluded  No calibration measures reported  No external validation |
| Du et al, 2022 [38] | Retrospective cohort  Single center  University hospital in China  Inclusion period: Pre-lockdown January to June 2019 and post-lockdown January to May 2020  Follow-up period: NR | n = 46 people admitted with DFU (23 + 23)  Mean age: >66 years  76% male  Mean diabetes duration: >11 years  Inclusion criteria: DFU with IWGDF guidelines diagnostic criteria; WIfI grade 1-3, class 1-3 ischemia, and class 1-3 infection requiring emergency admission | Development (train) (n=10 without outcome and 3 with outcome randomly selected):  6 different models  logistic regression  support vector machine  random forest  GBDT  artificial neural network  XGBoost  Internal validation (test) (n=8 without outcome and 2 with outcome randomly selected):  3-fold cross-validation | 31 variables tested, being the variables with more weight  Age, diabetes duration, diabetes treatment, nephropathy, education level, smoking pack-years, HDL, WBC, blood potassium, blood sodium, pre-hospital delay, WIfI wound, Ischemia | LEA | LEA not reported  Logistic regression  AUC: 0.76  Accuracy: 0.70  Sensitivity: 0.33  Specificity: 0.86  PPV: 0.50  NPV: 0.75  Support vector machine  AUC: 0.60  Accuracy: 0.70  Sensitivity: 0.00  Specificity: 1.00  PPV: 0.00  NPV: 0.70  Random forest  AUC: 0.67  Accuracy: 0.80  Sensitivity: 0.67  Specificity: 0.86  PPV: 0.67  NPV: 0.86  GBDT  AUC: 0.67  Accuracy: 0.70  Sensitivity: 0.33  Specificity: 0.86  PPV: 0.50  NPV: 0.75  XGBoost  AUC: 0.86  Accuracy: 0.80  Sensitivity: 0.67  Specificity: 0.86  PPV: 0.67  NPV: 0.86  Artificial neural network  AUC: 0.71  Accuracy: 0.70  Sensitivity: 0.33  Specificity: 0.86  PPV: 0.50  NPV: 0.75 | Retrospective  Single-center study  Patient follow-up period NR  Small sample  Models for LEA different between pre- and post-lockdown  No missing values reported  95% CI not reported  No calibration measures reported  No external validation |
| Hüsers et al, 2020 and 2022 [30,31] | Prospective cohort  Single-center  Wound care center in Germany  Inclusion period: 1^st^ June 2013 to 1^st^ July 2019  Follow-up period: 6 months | n = 237 participants with DFU  n=16 (6%) lost to follow up  Mean age: 66 years  84% male  Mean diabetes duration: NR  Exclusion criteria: other wounds (e.g.: venous or arterial leg ulcers) | Development (train):  Bayesian logistic regression model | 7 variables included in the model  Age, gender, PEDIS system classification composing variables (perfusion status, ulcer extent, ulcer depth, infection status and foot sensation) | Major LEA  Any LEA | 12.2% major LEA  31.6% LEA  Major LEA:  Cut off 0.28 (95% HDI 0.16 – 0.41)  AUC: 0.80 (95% HDI 0.78 – 0.80)  Sensitivity: 83% (95% HDI 71% – 93%)  Specificity: 66% (95% HDI 53% – 77%)  With no prior information:  AUC: 0.765 (95% HDI 0.725–0.779)  Any LEA, no prior information:  AUC: 0.793 (95% HDI 0.778–0.801)  Any and major LEA, prior information: AUC: 0.790 (95% HDI 0.774–0.802) | Single-center study  No missing values reported  Information provided does not allow model application  Outcome defined using ICD-9 codes  No calibration measures reported  No validation |

AUC: area under the curve; CI: confidence interval; DFU: diabetic foot ulcer; GBDT: gradient boosted decision trees; HbA1c: hemoglobin A1c; HDI: highest density intervals; HDL-C: high-density lipoprotein-cholesterol; ICD-9: International Classification of Diseases, 9th Revision; IWGDF: International Working Group on the Diabetic Foot; LASSO: least absolute shrinkage and selection operator; LDL-C: low-density lipoprotein-cholesterol; LEA: lower extremity amputation; LightGBM: light gradient-boosting machine; LoS: length of stay; NPV: negative predictive value; NR: not reported; OR: odds ratio; PAD: peripheral arterial disease; PEDIS: perfusion, extent, depth, infection, and sensation; PPV: positive predictive value; SHAP: shapley additive explanations; SMOTE: synthetic minority oversampling technique; USA: United States of America; WBC: white blood cell count; WIfI: wound, ischemia, and foot infection; XGBost: extreme gradient boosting.
