# Supplementary material for: Diabetic Foot Ulcer Classification Models Using Artificial Intelligence and Machine Learning Techniques: Systematic Review
Source: J Med Internet Res. 2025 Sep 24;27:e69408. doi: 10.2196/69408 (PMC12508669; doi:10.2196/69408)
Supplement: Multimedia Appendix 5 [file jmir_v27i1e69408_app5.doc]

**Multimedia Appendix 5.** Characteristics of the included studies organized by model development stage, study design, setting and sample size: mortality as outcome.

| **Reference** | **Study design, setting and follow-up** | **Study population and characteristics** | **Development/ validation** | **Variables assessed** | **Primary and secondary outcomes** | **Results** | **Comments** |
| --- | --- | --- | --- | --- | --- | --- | --- |
| Austin et al, 2022 [34] | Retrospective cohort  Multicenter  Fee-for-service Medicare database, USA  Inclusion period: 2015  Follow-up period: 6 months | n = 88,898 people with DFU and PAD  Mean age: 77 years  53 % female  Mean diabetes duration: NR  Inclusion criteria: newly diagnosed with concomitant PAD and diabetes; USA residents; age between 65 and 95; Medicare beneficiaries for 1 year following index date (first claim containing first diabetes-related ICD-9 or 10 code); ulcer diagnosis during first 6 months of the index year; outcome-free (alive, no reinterventions or amputations) for at least 6 months after index date | Development (train):  2/3 of sample  Logistic regression; random forest, using a data set randomly divided into training (2/3) and testing (1/3) groups  Calibration of logistic regression model: McFadden R^2^  Internal validation (test)  1/3 of sample | 9 variables tested  Charlson comorbidity index, sex, race, age at diagnosis, Medicare-Medicaid dual-eligibility status, urban/rural indicator, HbA1c, foot exam, vascular imaging study | Death | Mortality rate was 4.5%  Logistic regression  McFadden R^2^:0.046  Out-of-bag error rate:68%  Random forest  Out-of-bag error rate: 30% | Retrospective  Insurance based database  No missing values reported  Excluded lost to follow-up  Information provided does not allow model application  Not described how sample was split for train and test  No external validation  95% CI not reported |
| Du et al, 2022 [38] | Retrospective cohort  Single center  University hospital in China  Inclusion period: Pre-lockdown January to June 2019 and post-lockdown January to May 2020  Follow-up period: NR | n = 46 people admitted with DFU (23 + 23)  Mean age: >66 years  76% male  Mean diabetes duration: >11 years  Inclusion criteria: DFU with IWGDF guidelines diagnostic criteria; WIfI grade 1-3, class 1-3 ischemia, and class 1-3 infection requiring emergency admission | Development (train) (n=10 without outcome and 3 with outcome randomly selected):  6 different models  logistic regression  support vector machine  random forest  GBDT  artificial neural network  XGBoost  Internal validation (test) (n=8 without outcome and 2 with outcome randomly selected):  3-fold cross-validation | 31 variables tested, being the variables with more weight  For mortality:  Infection (not foot related), age, foot infection, PAD, WBC, education level, diabetes treatment, LDL, cerebral infarction, HDL | Mortality | Mortality 0% pre- vs. 17.4% post-lockdown  Logistic regression  AUC: 0.81  Accuracy: 0.70  Sensitivity: 0.50  Specificity: 0.75  PPV: 0.33  NPV: 0.86  Support vector machine  AUC: 0.56  Accuracy: 0.80  Sensitivity: 0.00  Specificity: 1.00  PPV: 0.00  NPV: 0.80  Random forest  AUC: 0.88  Accuracy: 0.80  Sensitivity: 0.50  Specificity: 0.88  PPV: 0.50  NPV: 0.88  GBDT  AUC: 0.88  Accuracy: 0.90  Sensitivity: 1.00  Specificity: 0.88  PPV: 0.67  NPV: 1.00  XGBoost  AUC: 0.94  Accuracy: 0.90  Sensitivity: 1.00  Specificity: 0.88  PPV: 0.67  NPV: 1.00  Artificial neural network  AUC: 0.69  Accuracy: 0.70  Sensitivity: 0.50  Specificity: 0.75  PPV: 0.33  NPV: 0.86 | Retrospective  Single-center study  Patient follow-up period NR  Small sample  No missing values reported  95% CI not reported  No calibration measures reported  No external validation |

AUC: area under the curve; CI: confidence interval; DFU: diabetic foot ulcer; GBDT: gradient boosted decision trees; HbA1c: hemoglobin A1c; HDL-C: high-density lipoprotein-cholesterol; ICD-9: International Classification of Diseases, 9th Revision; IWGDF: International Working Group on the Diabetic Foot; LDL-C: low-density lipoprotein-cholesterol; NPV: negative predictive value; NR: not reported; PAD: peripheral arterial disease; PPV: positive predictive value; USA: United States of America; WBC: white blood cells count; WIfI: wound, ischemia, and foot infection; XGBost: extreme gradient boosting.
