# Supplementary material for: Diabetic Foot Ulcer Classification Models Using Artificial Intelligence and Machine Learning Techniques: Systematic Review
Source: J Med Internet Res. 2025 Sep 24;27:e69408. doi: 10.2196/69408 (PMC12508669; doi:10.2196/69408)
Supplement: Multimedia Appendix 6 [file jmir_v27i1e69408_app6.doc]

**Multimedia Appendix 6.** Distribution of clinical variables included in the final models by categories having healing as outcome.

| **Variable categories** | | **Variables** | **Studies [References]** |
| --- | --- | --- | --- |
| Demographic characteristics | | Sex^a^ | 3 [28,35,36] |
|  |  | Age | 2 [35,36] |
|  |  | Center code | 1 [36] |
|  |  | Ethnicity/race | 1 [35] |
|  |  | Primary insurance | 1 [36] |
| Medical history | Comorbidities | Diabetes-related retinopathy | 2 [28,35] |
|  |  | Charlson comorbidity index | 1 [35] |
|  |  | CKD stage | 1 [35] |
|  |  | History of amputation | 1 [39] |
|  |  | Palliative care | 1 [36] |
|  |  | Peripheral arterial disease | 1 [28] |
|  |  | Smoking history | 1 [28] |
|  | Drugs | Oral antihyperglycemic drugs/insulin/canagliflozin | 2 [28,35] |
|  |  | Allopurinol | 1 [35] |
|  |  | Antihypertensives | 1 [35] |
|  |  | Aspirin | 1 [35] |
|  |  | Heparin/warfarin/Xa inhibitors | 1 [35] |
|  |  | Immunosuppressants/oral steroids | 1 [35] |
|  |  | NSAIDs | 1 [35] |
|  | Others | Body mass index | 2 [33,35] |
|  |  | Body surface area | 1 [35] |
| Laboratory data | | CRP | 3 [28,35,39] |
|  |  | Albumin | 2 [28,35] |
|  |  | ESR | 2 [35,39] |
|  |  | Creatinine | 1 [28] |
|  |  | HbA1c | 1 [35] |
|  |  | Lymphocyte count | 1 [35] |
|  |  | Pre-albumin | 1 [35] |
|  |  | Random blood glucose | 1 [28] |
| Foot related characteristics | | Wound area | 4 [28,33,36,39] |
|  |  | Probe-to-bone test | 2 [35,39] |
|  |  | Temperature | 2 [36,39] |
|  |  | Wound depth | 2 [35,36] |
|  |  | Wound duration | 2 [33,39] |
|  |  | Wound length | 2 [35,36] |
|  |  | Wound width | 2 [35,36] |
|  |  | ABI | 1 [39] |
|  |  | Ankle systolic pressure measured/ toe systolic pressure measured | 1 [35] |
|  |  | Atrophie blanche | 1 [36] |
|  |  | Brawny induration | 1 [36] |
|  |  | Callus | 1 [36] |
|  |  | Clustered wound | 1 [36] |
|  |  | Complex care | 1 [36] |
|  |  | Crepitus | 1 [36] |
|  |  | Cyanosis | 1 [36] |
|  |  | Delayed recurrence | 1 [36] |
|  |  | Dorsalis pedis pulse measured/ posterior tibial pulse measured | 1 [35] |
|  |  | Duration of care | 1 [36] |
|  |  | Ecchymosis | 1 [36] |
|  |  | Edema | 1 [36] |
|  |  | Epithelization | 1 [36] |
|  |  | Erythema | 1 [36] |
|  |  | Excoriation | 1 [36] |
|  |  | Exposed bone | 1 [36] |
|  |  | Exposed joint | 1 [36] |
|  |  | Exposed muscle | 1 [36] |
|  |  | Exposed tendon | 1 [36] |
|  |  | Exudate | 1 [36] |
|  |  | Fluctuance | 1 [36] |
|  |  | Foot | 1 [35] |
|  |  | Friable | 1 [36] |
|  |  | Granulation amount | 1 [36] |
|  |  | Granulation quality | 1 [36] |
|  |  | Hemosiderosis | 1 [36] |
|  |  | Infection | 1 [35] |
|  |  | Margin | 1 [36] |
|  |  | MRI performed | 1 [35] |
|  |  | Number of wounds | 1 [36] |
|  |  | Offload | 1 [35] |
|  |  | Pallor | 1 [36] |
|  |  | Pending amputation on presentation | 1 [36] |
|  |  | Presence of blood flow in the Doppler probe | 1 [39] |
|  |  | Previous wound count | 1 [36] |
|  |  | Rash | 1 [36] |
|  |  | Result of accident | 1 [36] |
|  |  | Rubor | 1 [36] |
|  |  | Slough | 1 [36] |
|  |  | Tc99 bone scan performed | 1 [35] |
|  |  | Tenderness on palpation | 1 [36] |
|  |  | Thickness | 1 [36] |
|  |  | Total contact cast use | 1 [35] |
|  |  | Transcutaneous oxygen pressure | 1 [35] |
|  |  | Tunneling | 1 [36] |
|  |  | Undermining | 1 [36] |
|  |  | UTSA stage/ UTSA grade [ischemia]/ wound grade | 1 [35] |
|  |  | Wound location | 1 [36] |
|  |  | Wound recurrence | 1 [36] |
|  |  | Wound type | 1 [36] |
|  |  | X-ray performed | 1 [35] |

ABI: ankle-brachial index; CKD: chronic kidney disease; CRP: C-reactive protein; ESV: erythrocyte sedimentation rate; HbA1c: hemoglobin A1c; MRI: magnetic resonance imaging; NSAIDs: non-steroidal anti-inflammatory drugs; Tc99: technetium-99 bone scan; UTSA: University of Texas Santo Antonio staging.

^a^Both ‘sex’ and ‘gender’ are mentioned in the articles; for clarity purposes, only the term ‘sex’ was used.
