# Supplementary material for: Diabetic Foot Ulcer Classification Models Using Artificial Intelligence and Machine Learning Techniques: Systematic Review
Source: J Med Internet Res. 2025 Sep 24;27:e69408. doi: 10.2196/69408 (PMC12508669; doi:10.2196/69408)
Supplement: Multimedia Appendix 7 [file jmir_v27i1e69408_app7.doc]

**Multimedia Appendix 7.** Distribution of clinical variables included in the final models by categories having lower extremity amputation as outcome.

| **Variable categories** | | **Variables** | **Studies [References]** |
| --- | --- | --- | --- |
| Demographic characteristics | | Age | 5 [30,31,34,37,38,40] |
|  |  | Sex^a^ | 3 [30,31,34,37] |
|  |  | Education level | 1 [38] |
|  |  | Insurance status | 1 [34] |
|  |  | Race | 1 [34] |
|  |  | Urban/rural | 1 [34] |
| Medical history | Comorbidities | Diabetes duration | 3 [29,37,38] |
|  |  | Smoking history | 3 [29,37,38] |
|  |  | DFU history | 2 [29,37] |
|  |  | Diabetes-related nephropathy | 2 [37,38] |
|  |  | Peripheral vascular disease | 2 [32,37] |
|  |  | Arterial occlusion | 1 [37] |
|  |  | Cardiovascular disease | 1 [29] |
|  |  | Cerebral infarction | 1 [37] |
|  |  | Charlson comorbidity index | 1 [34] |
|  |  | Coronary heart disease | 1 [37] |
|  |  | Diabetic neuropathy | 1 [37] |
|  |  | Diabetic retinopathy | 1 [37] |
|  |  | Heart failure | 1 [37] |
|  |  | History of diabetes | 1 [40] |
|  |  | Hypertension | 1 [37] |
|  |  | Other comorbidities | 1 [40] |
|  |  | Peripheral arterial disease | 1 [29] |
|  |  | Prior amputation | 1 [37] |
|  |  | Systemic infection | 1 [32] |
|  | Drugs | Antihyperglycemic drugs/insulin | 2 [37,38] |
|  | Others | Pre-hospital delay | 2 [37,38] |
|  |  | Weight/weight loss | 2 [32,40] |
|  |  | Body mass index | 1 [37] |
|  |  | Days admitted | 1 [40] |
|  |  | Duration of symptoms | 1 [40] |
| Laboratory data | | Albumin | 3 [29,37,40] |
|  |  | Creatinine | 3 [29,37,40] |
|  |  | HbA1c | 3 [34,37,40] |
|  |  | Random blood glucose | 3 [29,37,40] |
|  |  | HDL | 2 [37,38] |
|  |  | Hemoglobin | 2 [37,40] |
|  |  | Potassium | 2 [37,38] |
|  |  | Sodium | 2 [37,38] |
|  |  | WBC/percentage of neutrophils | 2 [29,37] |
|  |  | Bilirubin | 1 [40] |
|  |  | CRP | 1 [29] |
|  |  | Culture report growth | 1 [40] |
|  |  | LDL | 1 [37] |
|  |  | Prothrombin time | 1 [40] |
|  |  | Total cholesterol | 1 [37] |
|  |  | Triglyceride | 1 [37] |
| Foot related characteristics | | Gangrene | 2 [32,37] |
|  |  | Vascular imaging study/arterial doppler flow | 2 [34,40] |
|  |  | WIfI classification system | 2 [29,37] |
|  |  | Foot exam | 1 [34] |
|  |  | Foot X-ray | 1 [40] |
|  |  | Ischemia | 1 [38] |
|  |  | Osteomyelitis | 1 [32] |
|  |  | PEDIS classification system | 1 [30,31] |
|  |  | Ulcer grade | 1 [40] |
|  |  | Wagner classification system | 1 [37] |

CRP: C-reactive protein; DFU: diabetic foot ulcer; HbA1c: hemoglobin A1c; HDL: high-density lipoprotein; LDL: low-density lipoproteins; PEDIS: perfusion status, ulcer extent, ulcer depth, infection status and foot sensation; WBC: white blood cells count; WiFI: wound, ischemia, and foot infection.

^a^Both ‘sex’ and ‘gender’ are mentioned in the articles; for clarity purposes, only the term ‘sex’ was used.
