# Supplementary material for: Diabetic Foot Ulcer Classification Models Using Artificial Intelligence and Machine Learning Techniques: Systematic Review
Source: J Med Internet Res. 2025 Sep 24;27:e69408. doi: 10.2196/69408 (PMC12508669; doi:10.2196/69408)
Supplement: Multimedia Appendix 8 [file jmir_v27i1e69408_app8.doc]

**Multimedia Appendix 8.** Distribution of clinical variables included in the final models by categories having mortality as outcome.

| **Variable categories** | | **Variables** | **Studies [References]** |
| --- | --- | --- | --- |
| Demographic characteristics | | Age | 2 [34,38] |
|  |  | Education level | 1 [38] |
|  |  | Insurance status | 1 [34] |
|  |  | Race | 1 [34] |
|  |  | Sex | 1 [34] |
|  |  | Urban/rural | 1 [34] |
| Medical history | Comorbidities | Cerebral infarction | 1 [38] |
|  |  | Charlson comorbidity index | 1 [34] |
|  |  | Infection [not foot related] | 1 [38] |
|  |  | Peripheral arterial disease | 1 [38] |
|  | Drugs | Diabetes treatment | 1 [38] |
| Laboratory data | | HbA1c | 1 [34] |
|  |  | HDL | 1 [38] |
|  |  | LDL | 1 [38] |
|  |  | WBC | 1 [38] |
| Foot related characteristics | | Foot exam | 1 [34] |
|  |  | Foot infection | 1 [38] |
|  |  | Vascular imaging study | 1 [34] |

HbA1c: hemoglobin A1c; HDL: high-density lipoprotein; LDL: low-density lipoprotein; WBC: white blood cells count.
