# Supplementary material for: Diabetic Foot Ulcer Classification Models Using Artificial Intelligence and Machine Learning Techniques: Systematic Review
Source: J Med Internet Res. 2025 Sep 24;27:e69408. doi: 10.2196/69408 (PMC12508669; doi:10.2196/69408)
Supplement: Multimedia Appendix 9 [file jmir_v27i1e69408_app9.doc]

**Multimedia Appendix 9. QUIPS tool results of included studies.**

| **Author, year** | **Study participation** | **Study attrition** | **Prognostic factor measurement** | **Outcome measurement** | **Study confounding** | **Statistical analysis and reporting** | **Overall RoB** |
| --- | --- | --- | --- | --- | --- | --- | --- |
| Austin et al, 2022 [34] | Moderate | High | Moderate | Low | Low | Low | **High** |
| Du et al, 2022 [38] | Moderate | High | High | Low | Low | Low | **High** |
| Hüsers et al, 2020 and 2022 [30,31] | Moderate | High | Moderate | Low | Low | Low | **High** |
| Jung et al, 2016 [36] | Moderate | High | High | High | Low | Low | **High** |
| Kasbekar et al, 2017 [40] | High | High | High | Low | Low | Low | **High** |
| Kim et al, 2020 [35] | Moderate | High | Moderate | Moderate | Low | Low | **High** |
| Margolis et al, 2022 [33] | Moderate | Low | High | Moderate | Low | Low | **High** |
| Poradzka and Czupryniak, 2023 [39] | Moderate | Low | High | Moderate | Low | Low | **High** |
| Stefanopoulos et al, 2024 [32] | Moderate | High | High | Low | Low | Low | **High** |
| Wang et al, 2022a and b [28,29] | Moderate | High | Moderate | Low | Low | Low | **High** |
| Xie et al, 2022 [37] | High | High | High | Low | Low | Low | **High** |

RoB: risk of bias. A darker tone of the cell represents a higher risk of bias.
