# Supplementary material for: Diabetic Foot Ulcer Classification Models Using Artificial Intelligence and Machine Learning Techniques: Systematic Review
Source: J Med Internet Res. 2025 Sep 24;27:e69408. doi: 10.2196/69408 (PMC12508669; doi:10.2196/69408)
Supplement: Multimedia Appendix 10 [file jmir_v27i1e69408_app10.doc]

**Multimedia Appendix 10. PROBAST tool results of included studies.**

| **Author, year** | **RoB** | | | | **Applicability** | | | **Overall** | |
| --- | --- | --- | --- | --- | --- | --- | --- | --- | --- |
|  | **Participants** | **Predictors** | **Outcome** | **Analysis** | **Participants** | **Predictors** | **Outcome** | **RoB** | **Applicability** |
| Austin et al, 2022 [34] | + | ? | + | - | - | ? | + | **-** | **-** |
| Du et al, 2022 [38] | + | ? | ? | - | - | ? | + | **-** | **-** |
| Hüsers et al, 2020 and 2022 [30,31] | + | + | + | - | + | + | + | **-** | **+** |
| Jung et al, 2016 [36] | ? | ? | ? | - | ? | ? | - | **-** | **-** |
| Kasbekar et al, 2017 [40] | + | ? | ? | - | + | ? | + | **-** | **?** |
| Kim et al, 2020 [35] | - | ? | - | - | - | ? | - | **-** | **-** |
| Margolis et al, 2022 [33] | ? | ? | - | - | - | ? | - | **-** | **-** |
| Poradzka and Czupryniak, 2023 [39] | + | + | - | - | + | + | - | **-** | **-** |
| Stefanopoulos et al, 2024 [32] | ? | ? | ? | - | ? | ? | + | **-** | **?** |
| Wang et al, 2022a [28] | - | ? | + | - | - | ? | + | **-** | **-** |
| Wang et al, 2022b [29] |  |  | ? |  |  |  |  |  |  |
| Xie et al, 2022 [37] | + | ? | ? | - | + | ? | + | **-** | **?** |

RoB: risk of bias; +: low RoB or low concern regarding applicability; -: high RoB or high concern regarding applicability; ?: unclear RoB or unclear concern regarding applicability. A darker tone of the cell represents a higher risk of bias.
